# Introduction to Supercapacitors

**DOI:** 10.1039/d3na90074f

**Published:** 2023-07-24

**Authors:** Zhaojun Han, Ruopian Fang, Dewei Chu, Da-Wei Wang, Kostya (Ken) Ostrikov

**Affiliations:** a School of Chemical Engineering, The University of New South Wales Kensington NSW 2052 Australia zhaojun.han@unsw.edu.au; b CSIRO Manufacturing 36 Bradfield Road, Lindfield NSW 2070 Australia zhaojun.han@csiro.au; c School of Materials Science and Engineering, The University of New South Wales Kensington NSW 2052 Australia d.chu@unsw.edu.au; d School of Chemistry and Physics, QUT Centre for Materials Science, Queensland University of Technology (QUT) Brisbane QLD 4000 Australia

## Abstract

Guest editors Zhaojun Han, Ruopian Fang, Dewei Chu, Da-Wei Wang and Kostya (Ken) Ostrikov, introduce this *Nanoscale Advances* themed issue on supercapacitors.
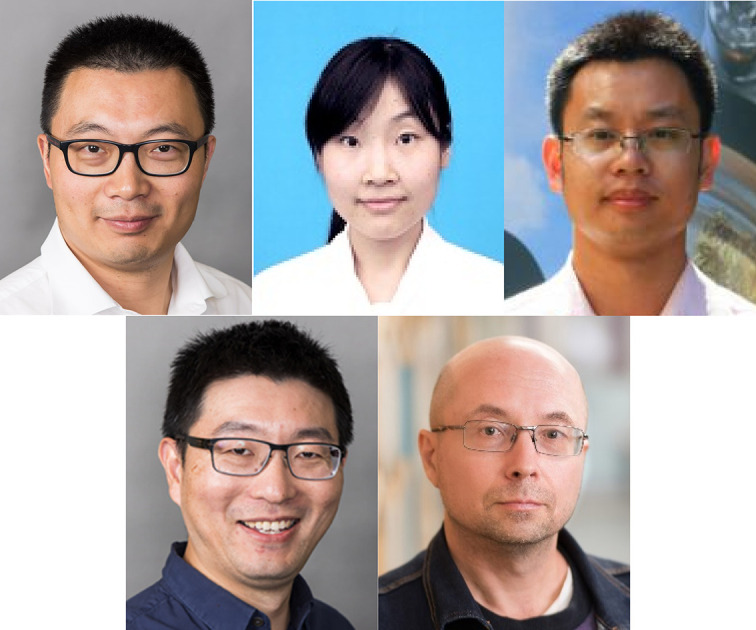

Our society is rapidly transitioning to clean energy to better manage climate change and keep the global temperature rise below 1.5 °C within this century. Electrochemical energy storage devices such as batteries and supercapacitors are expected to play a key role in this transition. In recent years, there has already been a boost in the applications of lithium-ion batteries in electric vehicles and stationary grid electricity storage. Supercapacitors (also commonly referred to as electrochemical capacitors), which store electric charges through either static adsorption (*i.e.*, electric double-layer capacitance) or redox reaction (*i.e.*, pseudocapacitance) mechanisms, have strong potential to complement or even replace batteries in many areas of applications.

Supercapacitors exhibit unique features of high power density, fast charging/discharging rates, long lifespan and safe operation, which can bring many benefits such as reduced charging time from hours to minutes, increased renewable electricity reliability, reduction in waste, and use of environmentally friendly materials. Commercial devices utilizing supercapacitors are widely used in regenerative braking systems for vehicles and elevators, computer memory backup, electricity grid stabilization, electric buses, consumer electronics like Samsung's stylus pen, to name a few.

The major challenge in supercapacitors is that conventional devices have a relatively low energy density of 5–20 W h kg^−1^, which is about 20 to 40 times lower than that of lithium-ion batteries (100–265 W h kg^−1^). Significant research effort has therefore been devoted to improving the energy density without compromising the excellent power density (typically in the range of 1–10 kW kg^−1^). As energy density *E* is given by *E* = 1/2 × *CV*^2^, where *C* is capacitance and *V* is the potential window, developing new electrode materials with high capacitance *C* or widening the potential window *V* using novel electrolytes, represents a sensible way to enhance energy density. A broad range of electrode materials have been developed in recent years, including activated carbon, carbon nanotubes (CNTs), graphene, MXene, transition metal dichalcogenides (TMDs), layered double hydroxides (LDHs), metal oxides, and conductive polymers. Particularly for two-dimensional (2D) layered materials, ion intercalation between the layers can provide a fast-charging mechanism with good capacity. In combination with structural engineering such as doping and pore size tuning, the gravimetric and volumetric capacitance of 2D electrode materials can be greatly enhanced. For instance, using a vacuum assisted assembly and electrostatic attraction between graphene oxide (GO) and exfoliated graphene (EG), a film with precisely adjusted interlayer spacing matching the size of electrolyte ions could be fabricated, resulting in highly efficient pore utilization and high volumetric capacitance.^1^

Extending the operational voltage *V* of supercapacitors is another effective approach to improve the energy density, as *E* scales with *V*^2^. The voltage is primarily determined by electrolytes, which include aqueous, organic, and ionic liquid electrolytes. While organic electrolytes are the main choice for commercial supercapacitor devices, room-temperature ionic liquids give the highest voltage (beyond 4 V). Owing to their environmentally benign nature, an increasing trend has also been seen in developing aqueous supercapacitors with high voltage using water in salt or asymmetric configurations.^2^

It is thus our great pleasure to introduce this themed collection on supercapacitors to the multidisciplinary research community. The collection covers research papers on electrode materials and high voltage from basic concepts to applications. For example, Shah *et al.* report a facile wet-chemical synthesis of FeSe_2_/TiO_2_ nanocomposites and constructed an asymmetric supercapacitor which exhibited a wide voltage of 2.3 V in an aqueous solution (https://doi.org/10.1039/D2NA00842D). The asymmetric supercapacitor demonstrated a high energy density of ∼70 W h kg^−1^ with outstanding long-term cycling stability and rate performance. In their minireview, Doo *et al.* systematically examined the advances in electrical double layer capacitor (EDLC) to achieve a high operating voltage (https://doi.org/10.1039/D2NA00863G). The article describes topics ranging from materials and electrolytes to long-term device perspectives for next-generation supercapacitor-based energy storage systems.

Moreover, recent research has shown exciting progress in developing multifunctional supercapacitors, which not only store electrical energy but also offer additional functionalities. Zhou *et al.* present a comprehensive review on multifunctional supercapacitors with mechanical, thermal, electronic, optical, magnetic, and energy harvesting capabilities.^3^ A typical multifunctional supercapacitor is the structural supercapacitor, which integrates energy storage capabilities into composite structures, eliminating the extra weight associated with conventional storage systems. Structural supercapacitors may thus represent the future of ‘massless’ energy solutions to turn structural components such as car body or airplane fuselage into a new avenue of energy storage. In addition, supercapacitors that can operate under extremely high or low temperatures are attractive for applications under harsh conditions. In this themed collection, Mishukova *et al.* reported the facile fabrication of graphene-based high-performance micro-supercapacitors operating at a high temperature of 150 °C (https://doi.org/10.1039/D1NA00220A), which held promise for integrating thermally stable energy storage devices at a reduced size for applications such as implantable biomedical devices and sensors.

*Nanoscale Advances* is an international gold open access journal which publishes high-quality research across the breadth of nanoscience and nanotechnology. It is undoubtedly one of the best places for publishing materials innovations in supercapacitors and other energy storage devices. We are honored to have this opportunity to present this themed collection on supercapacitors and appreciate the excellent support received from the editorial team. We believe this topic is timely and look forward to seeing more relevant research published in the near future.

## Supplementary Material
